# Focal Adhesion Kinase Binds to the HPV E2 Protein to Regulate Initial Replication after Infection

**DOI:** 10.3390/pathogens12101203

**Published:** 2023-09-28

**Authors:** Leny Jose, Jessica Gonzalez, Emma Kessinger, Elliot J. Androphy, Marsha DeSmet

**Affiliations:** 1 Department of Dermatology, Indiana University School of Medicine, Indianapolis, IN 46202, USA; lenyjose@iu.edu (L.J.); emmakessinger@gmail.com (E.K.); eandro@iu.edu (E.J.A.); 2 Department of Microbiology and Immunology, Indiana University School of Medicine, Indianapolis, IN 46202, USA; jekagonz@indiana.edu

**Keywords:** HPV, focal adhesion kinase, replication

## Abstract

Human papillomaviruses are small DNA tumor viruses that infect cutaneous and mucosal epithelia. The viral lifecycle is linked to the differentiation status of the epithelium. During initial viral infection, the genomes replicate at a low copy number but the mechanism(s) the virus uses to control the copy number during this stage is not known. In this study, we demonstrate that the tyrosine kinase focal adhesion kinase (FAK) binds to and phosphorylates the high-risk viral E2 protein, the key regulator of HPV replication. The depletion of FAK with a specific PROTAC had no effect on viral DNA content in keratinocytes that already maintain HPV-16 and HPV-31 episomes. In contrast, the depletion of FAK significantly increased HPV-16 DNA content in keratinocytes infected with HPV-16 quasiviruses. These data imply that FAK prevents the over-replication of the HPV genome after infection through the interaction and phosphorylation of the E2 protein.

## 1. Introduction

HPV replication is linked to the differentiation status of the infected epithelium [1,2]. Initial infection takes place in basal epithelial cells, where the viral genome replicates at a low copy number. The over-replication of episomes could lead to (1) increased viral E1, E2, E6, and E7 expression, triggering cell death [3,4,5,6,7,8] or (2) increased E2 levels to suppress Origin Recognition Complex Subunit 2 (ORC2) levels, origin firing, and to trigger cell senescence [9]. The mechanism the virus uses to preserve low copy number during viral replication upon infection is unclear [10]. PV genomes duplicate and partition to progeny cells in a cell-cycle-dependent manner in the basal and suprabasal cells [11,12]. This stage can be recreated in monolayer keratinocyte cultures that stably maintain viral DNA as episomes. In the final stage, amplification of viral episomes to hundreds of copies occurs as infected cells migrate into the upper epithelial strata in non-dividing and differentiating epithelial cells.

Basal levels of the viral E2 protein are required to initiate viral replication [13,14]. The E2 protein is composed of an approximately 220 amino acid transactivation domain (TAD), a hinge region, and a DNA binding domain (DBD). The E2 protein binds to inverted palindrome ACCN_6_GGT sequences at the viral origin of replication (ori) to support genome replication [15]. E2 is regulated by several post-translational modifications (PTMs) [16,17,18,19,20,21,22]; the biological functions of these E2 PTMs can be reviewed in reference [23]. We identified Pyk2, a nonreceptor tyrosine kinase and member of the focal adhesion kinase (FAK) subfamily, to suppress viral replication by binding and phosphorylating the HPV-16 and 31-E2 proteins at tyrosine 131 [24]. Pyk2 shares the same three-domain organization and sequence similarity to FAK, the other member of the FAK subfamily [25].

FAK is a tyrosine kinase involved in cell migration, adhesion, proliferation, and angiogenesis [26]. FAK is composed of an N-terminal FERM domain, a middle kinase domain, and a C-terminal Focal Adhesion Targeting (FAT) domain. FAK is thought to be involved in cytoplasmic kinase activation at cell adhesions and endosomes [27]. The FERM domain and the kinase domain contain the nuclear localization signal and the nuclear export signal for FAK nuclear shuttling [28,29]. There are numerous examples where FAK controls transcriptional activity (reviewed in reference [30]). For example, the FERM domain of FAK binds the N-terminal domain of p53 to inhibit its transcriptional activity [31]. FAK binds to MBD2 in the nucleus to release MBD2 and HDAC1 to promote muscle differentiation [32]. FAK also controls EZH2 transcription and nuclear localization by modulating p53 and E2F2/3 transcriptional activity [33].

Since FAK is expressed in keratinocytes [34], and we demonstrated that its close paralogue Pyk2 regulates viral replication through E2 association, we sought to determine if FAK could modulate viral replication. We describe here that FAK binds and phosphorylates high-risk HPV-16 and -31 E2 proteins. Unlike Pyk2, FAK depletion using a PROTAC had no effect on HPV-16 DNA content in cell lines that maintained HPV-16 episomes. However, after HPV-16 quasivirus infection of human keratinocytes, we found thatFAK depletion significantly increased HPV-16 DNA content in these lines. These results illustrate a novel mechanism by which FAK regulates HPV copy number immediately after infection to prevent over-replication.

## 2. Results

**Figure 1:**
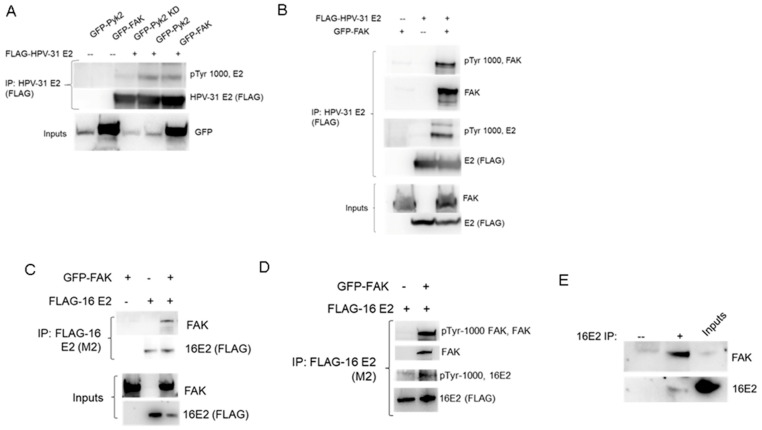
FAK interacts with and phosphorylates the PV E2 protein. (**A**) HEK293TT cells were transfected with GFP-Pyk2, GFP-Pyk2 KD (kinase dead, Y402F), GFP-FAK, and FLAG-HPV-31 E2 constructs. FLAG-HPV-31 E2 was immunoprecipitated with FLAG (M2) antibodies and complexes/inputs were blotted with FLAG (HPV-31 E2), GFP (Pyk2, FAK), and pTyr-1000 (pE2) antibodies. (**B**) HEK293TT cells were transfected with FLAG-HPV-31 E2 and GFP-FAK constructs and FLAG-HPV-31 E2 was immunoprecipitated with FLAG (M2) antibodies. Complexes were blotted with FLAG (HPV-31 E2), GFP (FAK), and pTyr-1000 (pFAK, pE2) antibodies. (**C**) HEK293TT cells were transfected with FLAG-HPV-16 E2 and GFP-FAK constructs and FLAG-HPV-16 E2 was immunoprecipitated with FLAG (M2) antibodies. Complexes were blotted with FLAG (HPV-16 E2), GFP (FAK), and (**D**) pTyr-1000 (pFAK, pE2) antibodies. (**E**) W12 cells were lysed and endogenous E2 immunoprecipitated with E2 antibodies (sheep anti-16E2). Proteins were blotted with FAK and HPV-16 E2 (TVG-261) antibodies. IgG sheep was used a negative control.

We previously demonstrated that Pyk2 was nuclear and that it bound the HPV-16 and 31-E2 proteins [24]. FAK has the same domain structure and 65% sequence similarity to Pyk2 [25], so we tested whether E2 associated with FAK. Like Pyk2, GFP-tagged FAK co-immunoprecipitated with FLAG-tagged HPV-31 E2 and phosphorylated HPV-31 E2 (Figure 1A,B). FLAG-HPV-16 E2 also co-immunoprecipitated with GFP-tagged FAK and phosphorylated HPV 16 E2 (Figure 1C,D). We also detected an interaction between endogenous HPV-16 E2 and FAK proteins in W12 cells (Figure 1E).

**Figure 2:**
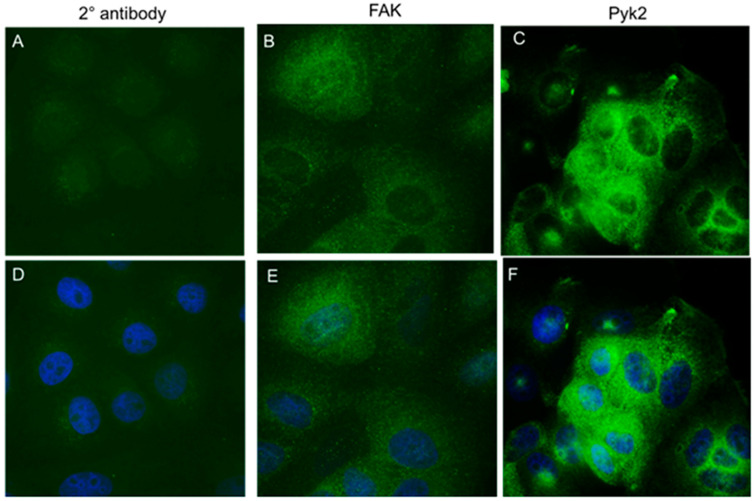
FAK protein is expressed in the nucleus of W12 cells. W12 cells (clone 20850) were stained for immunofluorescence with FAK and Pyk2 antibodies. Green (488), FAK (**B**,**E**) or Pyk2 (**C**,**F**); blue DAPI. Magnification ×40. Secondary antibodies alone were used as a negative control to determine fluorescence background (**A**,**D**).

The FAK protein is both nuclear and cytoplasmic. We performed immunofluorescence for FAK in the cervical HPV-16 keratinocyte dysplastic cell line W12 clone 20850. Although we observed that FAK and Pyk2 were predominantly cytoplasmic, we also observed nuclear staining (Figure 2).

**Figure 3:**
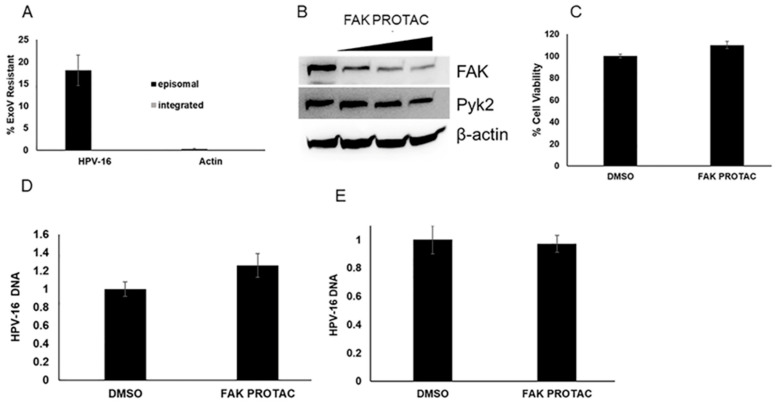
FAK protein degradation has no effect on HPV-16 viral replication in W12 cells. (**A**) Episomal status was confirmed in W12 cells (clone 20850). DNA from episomal and integrated W12 cells were subjected to exonuclease V digestion. Resistant HPV-16 DNA was quantified using PCR. Actin DNA served as a positive control for ExoV digestion. Values are means ± the SEM (n = 9). (**B**) BI-0319 was treated on episomal W12 cells as various concentrations (250 nM, 500 nM, 1 µM) for 48 h. Lysates were immunoblotted with FAK, Pyk2, and β-actin antibodies. (**C**) W12 episomal cells were treated with 1 µM BI-0319 (FAK PROTAC) for 72 h. Percent (%) cell viability, measured using MTT assay, was completed 72 h after treatment (n = 24). DNA from episomal W12 cells treated with control (DMSO) or 1 µM BI-0319 (FAK PROTAC) for 48 h (**D**) and 96 h (**E**) were analyzed with primers against the HPV-16 LCR and normalized to β-actin. Values are means ± the SEM (n = 8, n = 6).

To determine the effects of FAK on viral genome replication during the maintenance stage, we used W12 cells clone 20850. When cultured with J23T3 feeders, this cell line maintains the viral HPV-16 episomes. We confirmed the episomal status using an exonuclease V assay [35]. W12 cells with integrated HPV genomes were used as a control in this assay. As seen in Figure 3A, the W12 cells clone 20850 maintained HPV-16 episomes. We next treated the cells with either 250, 500, or 1000 nM of the FAK PROTAC, BI-0319. BI-0319 reduced FAK protein levels but not Pyk2 levels (Figure 3B). The treatment of W12 cells clone 20850 with 1 µM BI-0319 did not decrease cell viability at 72 h (Figure 3C) or change HPV-16 DNA content at 48 h (Figure 3D) or 96 h (Figure 3E).

**Figure 4:**
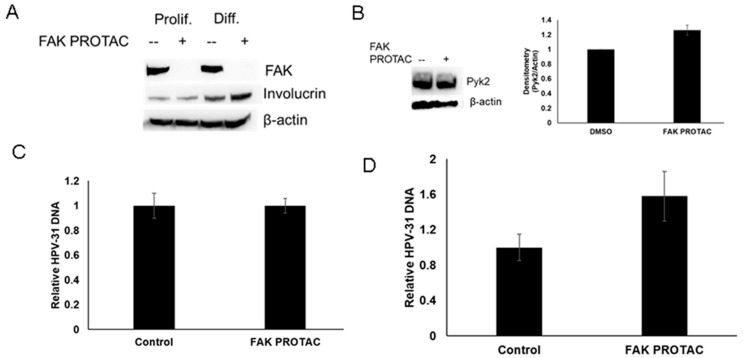
FAK protein degradation has no effect on HPV-31 DNA content in proliferating or differentiated CIN612 cells. (**A**) Episomal and differentiating CIN612 cells were treated with 1 µM BI-0319 for 72 h. Differentiation was induced with 2 mM CaCl_2_ for 72 h along with FAK PROTAC treatment. Lysates were immunoblotted with FAK and β-actin antibodies. (**B**) CIN612 cells were treated with 1 µM BI-0319 for 72 h. Lysates were immunoblotted with Pyk2 and β-actin antibodies. Blots were quantified using Image Lab. FAK PROTAC groups were normalized to DMSO groups. Values are means ±, n = 3. DNA from proliferating CIN612 proliferating (**C**) or differentiating (**D**) cells were treated with control (DMSO) or 1 µM BI-0319 (FAK PROTAC) for 72 h and analyzed with primers against the HPV-31 LCR and normalized to β-actin. Values are means ± the SEM (n = 9).

To determine whether FAK depletion affects viral replication during the amplification stage of viral lifecycle, we treated CIN612 cells which contain episomal HPV-31 with 2 mM calcium to induce keratinocyte differentiation. BI-0319 induced FAK degradation in both proliferating and differentiated cells (Figure 4A). BI-0319 did not decrease Pyk2 levels in CIN612 cells (Figure 3B). We did not observe any significant change in HPV-31 DNA content at 48 h in the proliferating cells while there was a modest increase in DNA content in the differentiated cells, but this was not significant (Figure 4C,D).

**Figure 5:**
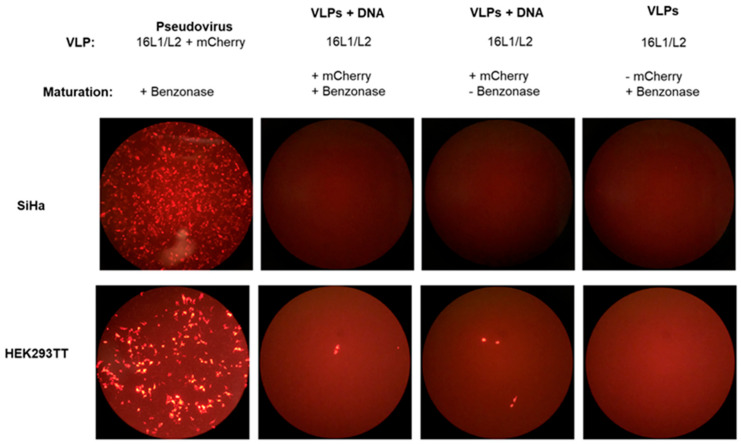
HPV-16 L1 and L2 PsV demonstrate robust infection but HPV-16 L1 and L2 capsids (VLPs) inefficiently deliver externally attached DNA. HEK293TT cells either transfected with (1) 16pSheLL and mCherry DNA and benzonase was added at virus maturation (Pseudovirus), (2) 16pSheLL DNA and mCherry DNA with benzonase was added at virus maturation, (3) 16pSheLL DNA and mCherry DNA was added at virus maturation, or (4) 16pSheLL DNA and benzonase was added at virus maturation. VLPs were added to SiHa or HEK293TT cells and mCherry was visualized 72 h post infection.

Our next goal was to determine if FAK influenced early viral replication initially after viral infection. In monolayer cell lines derived from HEK293, L1 and L2 self-assemble into virions and efficiently package co-transfected viral DNA (quasivirus) or any reporter DNA less than 8 Kb (pseudovirus, PsV) [36,37,38]. However, some studies suggest that these virus-like particles (VLPs) may also deliver extracellular DNA to human cells through L2-DNA binding [39,40]. First, we verified that, under our experimental conditions, DNA was not hitchhiking into the cells along with the VLPs with empty capsids, which would not reflect natural HPV infection. To achieve this, HPV-16 L1/L2 VLPs with externally attached mCherry DNA were treated with and without benzonase endonuclease to degrade non-encapsulated DNA during viral maturation. As seen in Figure 5, mCherry protein was detected in low levels in the HPV-16 VLPs with benzonase sensitive mCherry DNA in both SiHa and HEK293TT cells compared to the mCherry pseudovirus. This suggests that HPV-16 L1/L2 PsV promotes robust infection but cannot efficiently deliver externally attached DNA.

**Figure 6:**
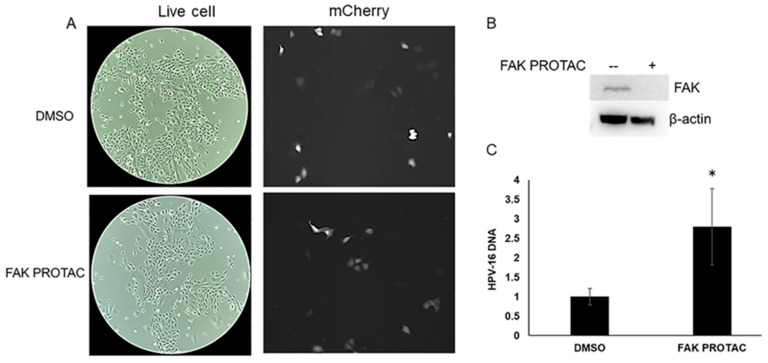
FAK protein degradation increases HPV-16 DNA copy number after initial viral infection. (**A**) NIKS cells were infected with mCherry PsV and treated with 1 µM FAK PROTAC (BI-0319) at infection for 48 h. (**B**) Lysates were immunoblotted with FAK and β-actin antibodies. (**C**) DNA from NIKS cells infected with HPV-16neo quasiviruses and treated with control (DMSO) or 1 µM BI-0319 (FAK PROTAC) for 72 h were analyzed with primers against the HPV-16 LCR and normalized to β-actin. Values are means ± the SEM (n = 9). * *p* < 0.05.

We packaged HPV-16neo (L1 and L2 genes were replaced with a neomycin cassette) DNA into HPV-16 L1/L2 capsids (VLPs). NIKS cells were infected with mCherry pseudovirus and HPV-16 quasivirus at the time of BI-0319 treatment. We did not observe differences in mCherry protein levels after infection (Figure 6A) with FAK depletion (Figure 6B) using fluorescence microscopy. Next, we measured HPV-16 DNA content 72 h post infection in NIKS cells. We observed a significant increase (>2.5 fold) in HPV-16 DNA content in cells treated with the FAK PROTAC compared to the control (Figure 6C).

## 3. Discussion

In this study, we demonstrate the importance of FAK activity on initial HPV replication. Similar to previously studied Pyk2, FAK binds to and increases HPV-E2 tyrosine phosphorylation. Pyk2 siRNA and treatment with a FAK/PYK2 dual specificity inhibitor, PF-431396, significantly increased HPV DNA content in cell lines that maintain viral episomes [24]. However, we did not observe the same effect following FAK degradation in these lines. One explanation for this may be related to the expression levels of these kinases in basal vs. upper-level keratinocytes. Data from GTExPortal show that FAK gene (PTK2) is expressed at 13.43 and 14.82 transcripts per million (TPM) compared to the Pyk2 gene (PTK2B) at 52.44 and 46.92 TPM in the non-sun exposed and sun exposed skin, respectively. The expression for FAK is at 20.93 and 21.21 TPM and Pyk2 at 23.92 and 25.85 TPM in endocervix and exocervix, respectively. The lower expression of FAK compared to Pyk2 may explain the difference in the responsiveness in the viral maintenance replicative stage.

Viral particles bind to heparan sulfate and activate FAK by phosphorylation at Y397 through the α6 integrin receptor [41]. The treatment of cells with the dual FAK/Pyk2 inhibitor TAE226 inhibited viral entry [41]. However, further studies revealed that FAK depletion using siRNA only decreased HPV-16 PsV infection by 20% compared to a 60% decrease by Pyk2 siRNA [42]. When we measured infection efficiency using mCherry PsVs, we did not visualize a significant difference in viral entry between the control and FAK PROTAC treated groups. Although small changes (<20%) cannot be accurately measured using this method, we still observed a >2.5-fold increase in HPV-16 DNA content post infection with FAK depletion.

We observed a modest increase, which was, however, not statistically significant, in viral genome content after FAK PROTAC treatment in differentiated CIN612 cells. Like during initial replication, replication during differentiation is not dependent on the host cell cycle. Our data suggest that FAK is an important regulator during bursts of replication. Calcium treatment-induced differentiation can increase FAK autophosphorylation and kinase activation [43]. These events may also lead to increased viral replication at this stage.

FAK mRNA and protein expression were significantly higher in cervical cancer cell lines when compared to moderate to well differentiated squamous cell carcinoma [44]. The treatment of these lines with FAK siRNA significantly reduced proliferation and invasion potential [44]. At this stage, cancer is driven by E6 and E7 expression due to viral integration. This mechanism is unlikely to be related to the HPV E2 protein since viral genome integration is likely to occur at the E2 loci in cervical cancers and in cervical cancer cell lines [45,46,47].

After infection in the basal epithelium, the virus must undergo replication at a low copy. E2 was one of the first transcripts expressed at 4 h post infection using native HPV-31 virions [48]. Viral replication was detected 48 h post infection using the quasivirus system described in this study [49]. Traditionally, it has been proposed that E2 must maintain low levels after initial infection, but this has not been directly tested. Here, we provide evidence that FAK phosphorylates the E2 protein. The degradation of FAK using a FAK-specific PROTAC significantly increased HPV DNA content 72 h post infection, suggesting that FAK is inhibitory to HPV replication at this stage. Our data provide evidence that, after viral infection, the virus utilizes the host kinase machinery to tightly control its DNA replication.

## 4. Materials and Methods

### 4.1. Plasmids and Antibodies

The following plasmids were used: pCDNA3-FLAG-HPV-31 and -16 E2, 16pSheLL (gift from J. Schiller), GFP-Pyk2 WT and KD [50], and pGFP-FAK [51]. The antibodies used included mouse anti-FLAG M2, anti-β-actin (Sigma, St. Louis, MO, USA), rabbit anti-Pyk2 (Sigma), rabbit anti-FAK (Cell signaling, Danvers, MA, USA), mouse anti-GFP (Santa Cruz Biotechnology, Dallas, TX, USA), sheep anti-E2 polyclonal serum [52], and rabbit anti-pTyr-1000 (Cell Signaling).

### 4.2. Cell Culture

All cell lines were maintained at 37 °C and 5% CO_2_. HEK293TT (from J. Schiller and C. Buck), J23T3 fibroblast feeders, HaCaT (from N. Fusenig), SiHa, and HEK293TTF (from R. Roden) were cultured in Dulbecco modified Eagle medium (DMEM; Life Technologies, Carlsbad, CA, USA) with 10% fetal bovine serum (FBS; Perk Serum) and penicillin/streptomycin (100 U/mL; Life Technologies). W12 cells (from P. Lambert), NIKS (from B. Allen-Hoffman), and CIN612 cells from (L. Laimins) were grown in F or E medium with mitomycin-C-treated J23T3 fibroblast feeders. Quasiviruses were produced as previously described [53] using the HPV-16neo genome where the L1 and L2 genes were replaced with a neomycin cassette [54] or pmCherry [55]. BI-0319 was a gift from OpnMe, and was diluted in DMSO at a stock of 250 µM. BI-0319 was added to W12 cells at various concentrations (250 nM, 500 nM, or 1 µM) for 48 or 96 h or to quasivirus-infected NIKS cells at 1 µM for 72 h.

### 4.3. Immunofluorescence

Cells were plated on a coverslip in 6-well dishes and immunofluorescence analysis was performed as previously described [56]. FAK (Cell Signaling) and Pyk2 antibodies (Sigma) were used at a 1:200 dilution. The secondary used was goat anti-rabbit 488 (Invitrogen) at a 1:1000 dilution. The secondary alone was used as a negative control for background fluorescence.

### 4.4. Co-Immunoprecipitations and Immunoblotting

Cells were transfected with PEI (2 mg/mL) and lysed, and immunoprecipitation and immunoblotting were performed as previously described [56] Inputs are 10% of the lysate.

### 4.5. HPV DNA Quantification

Cells were resuspended in PBS and isolated by using the DNeasy blood and tissue kit (Qiagen). For the exonuclease V digestion, 500 ng of DNA was either treated with exonuclease V (New England Biolabs) or left untreated for 4 h at 37 °C. The enzyme was inactivated at 70 °C for 20 min. Next, 50 ng of the digested or undigested DNA was quantified using real-time PCR. HPV-16/31 LCR and actin primers were previously described [9,53].{The percent of exonuclease V-resistant DNA was calculated as 2^–(Digested *CT* – Undigested *CT*)^ × 100.

### 4.6. Statistical Analysis

All experiments were repeated at least three independent times, except when stated otherwise, and results were averaged. The Student’s two-way *t*-test was used to find the significant value.

## Data Availability

Not applicable.
